# Conformational Changes in the Orai1 C-Terminus Evoked by STIM1 Binding

**DOI:** 10.1371/journal.pone.0128622

**Published:** 2015-06-02

**Authors:** Leidamarie Tirado-Lee, Megumi Yamashita, Murali Prakriya

**Affiliations:** Department of Pharmacology, Feinberg School of Medicine, Northwestern University, Chicago, IL, United States of America; Penn State Hershey College of Medicine, UNITED STATES

## Abstract

Store-operated CRAC channels regulate a wide range of cellular functions including gene expression, chemotaxis, and proliferation. CRAC channels consist of two components: the Orai proteins (Orai1-3), which form the ion-selective pore, and STIM proteins (STIM1-2), which form the endoplasmic reticulum (ER) Ca^2+^ sensors. Activation of CRAC channels is initiated by the migration of STIM1 to the ER-plasma membrane (PM) junctions, where it directly interacts with Orai1 to open the Ca^2+^-selective pores of the CRAC channels. The recent elucidation of the *Drosophila* Orai structure revealed a hexameric channel wherein the C-terminal helices of adjacent Orai subunits associate in an anti-parallel orientation. This association is maintained by hydrophobic interactions between the *Drosophila* equivalents of human Orai1 residues L273 and L276. Here, we used mutagenesis and chemical cross-linking to assess the nature and extent of conformational changes in the self-associated Orai1 C-termini during STIM1 binding. We find that linking the anti-parallel coiled-coils of the adjacent Orai1 C-termini through disulfide cross-links diminishes STIM1-Orai1 interaction, as assessed by FRET. Conversely, prior binding of STIM1 to the Orai1 C-terminus impairs cross-linking of the Orai1 C-termini. Mutational analysis indicated that a bend of the Orai1 helix located upstream of the self-associated coils (formed by the amino acid sequence SHK) establishes an appropriate orientation of the Orai1 C-termini that is required for STIM1 binding. Together, our results support a model wherein the self-associated Orai1 C-termini rearrange modestly to accommodate STIM1 binding.

## Introduction

Calcium (Ca^2+^) is a ubiquitous second messenger involved in the regulation of a wide assortment of cellular functions including gene expression, proliferation, and chemotaxis [1]. Within the Ca^2+^-signaling toolkit of non-excitable animal cells, the store-operated calcium (SOC) release-activated calcium (CRAC) channels, characterized for their activation by depletion of the endoplasmic reticulum (ER) Ca^2+^ stores, have emerged as a prominent route for Ca^2+^ mobilization [2]. The ensuing calcium signals mediate both short-term cellular functions, such as refilling of stores, as well as long-term functions, such as gene transcription [3]. Dysfunctions in CRAC channels are associated with a variety of pathologies such as severe combined immunodeficiency syndrome, myopathies, and bleeding disorders, highlighting their importance for human physiology [4]. More recently, evidence has begun to emerge implicating SOC machinery in a variety of pathophysiologies including cancer [5, 6], vascular diseases [7], and epilepsy [8]. Manipulation of CRAC channel function, therefore, offers the potential for development of new therapies for diseases of the immune system and beyond. However, many questions dealing with the mechanisms of how CRAC channels operate and function must be addressed to achieve this therapeutic potential.

The ER Ca^2+^ sensing stromal interaction molecules (STIMs) and the pore forming Orai proteins are the two fundamental components necessary and sufficient to reconstitute functional CRAC channels [9]. Following depletion of ER Ca^2+^ stores human STIM1 oligomerizes and migrates from the bulk ER to ER-PM (plasma membrane) junctions [10–12] where it traps diffusing human Orai1 channels in the overlying plasma membrane [13–16]. The minimal region of STIM1 required for binding to Orai1 and subsequent channel activation was mapped to ~ 100 amino acid segment (amino acids 342–448) commonly referred to as the CRAC activation domain (CAD) [9, 17–19]. CAD binds directly to the Orai1 C-terminus at a conserved, putative coiled-coil (CC) region (amino acids 268–291) and mutations disrupting the CC structure, such as L273S or L273D [20–22] and L276D [23], abrogate STIM1-Orai1 binding and channel activation [9, 17, 20, 23]. A second CAD binding site resides in the Orai1 N-terminus within the membrane proximal region of Orai1’s N-terminus (amino acids 73–87) recently shown to be a cytosolic extension of the ion conduction pathway [9, 17, 24]. Interestingly, deletions or mutations at this site give rise to non-conducting channels still capable of associating with STIM1, albeit to a lower extent than that seen with intact Orai1 [20, 25–29]. Emerging evidence suggests that these two sites function in both STIM1 binding and channel gating, pointing to a concerted mechanism rather than one where each site has a distinct role in either binding or activation [28].

**Fig 1:**
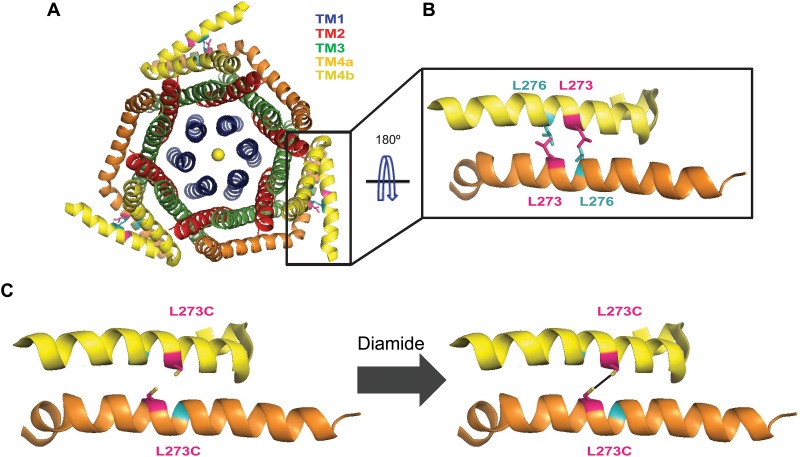
The Orai C-termini of adjacent monomers interact in an anti-parallel coiled-coil. (A) X-ray crystal structure of *Drosophila* Orai channel (PDB code ID 4HKR) viewed from the extracellular side. The channel displays six-fold symmetry within the pore formed by TM1 helices (blue) and the concentric layer formed by the TM2 (red) and TM3 (green) helices. At the outermost helical layer formed by the TM4 helices (orange and yellow), only a three-fold symmetry is observed due to the formation of an anti-parallel coiled-coil (box) between the cytosolic extensions of adjacent TM4 monomers. (B) Close-up of the hydrophobic pocket formed by putative interaction between L273 of human Orai1 (hot pink) in one monomer with L276 (teal) of the adjacent monomer. PyMol mutagenesis wizard was used to substitute a leucine residue at position 316 which is an isoleucine in the *Drosophila* structure. (C) Schematic of the experimental set-up. Exogenous cysteines were introduced at L273 or L276 individually. Diagram shows L273C mutations (created with PyMol mutagenesis wizard) on the adjacent TM4 monomers. If the introduced cysteines are close together, then addition of an oxidizing agent, such as diamide, could induce the formation of a disulfide bond.

Understanding the nature of the C-terminal CAD binding site of Orai has been the focus of many biochemical studies over the years. Early studies hypothesized that a negative patch on Orai1 (amino acids 263–285) [30–32] associates with a positive, polybasic patch within a coiled-coil CAD segment (amino acids 382–386) [31, 32]. However, further studies indicated that although electrostatic interactions may contribute to STIM1 interaction at the Orai1 C-terminus, other interactions, in particular involving hydrophobic coiled-coiled interactions, likely play a more critical role for the association of these two proteins [21, 30, 33]. The elucidation of a 3.35 Angstrom *Drosophila melanogaster* Orai1 crystal structure brought with it a multitude of new questions about the C-terminal binding site (Fig 1A) [24]. An unpredicted association was observed between the cytosolic C-termini of neighboring monomers via an anti-parallel coiled-coil mediated by the hydrophobic interaction of Ile316 (human L273) with Leu319 (human L276) (Fig 1B). Previous mutagenesis studies had shown that substitutions of these hydrophobic residues to residues that would lower the coiled-coil probability of the C-terminus, such as L273S [20, 21] and L276D [23], diminish STIM1 binding and disrupt channel activation. Hou *et al*. proposed that STIM1 binds to extended Orai1 C-termini that have straightened into the cytosol presenting six individual termini for STIM1 to interact with [24]. However, this model does not readily explain why the L273S and L276D mutations, which would be expected to disrupt Orai C-terminal self-association, impair STIM1-Orai1 binding [34].

Recently, an NMR complex structure was determined for a human STIM1 fragment (amino acids 312–387) and a human Orai1 C-terminal fragment (amino acids 272–292) [35]. The complex supports a STIM1-Orai1 interaction model featuring coiled-coil heterodimers of the two proteins mediated by both non-polar and polar interactions. Interestingly, this study notes that the general orientation of the Orai1 fragments bound to STIM1(312–387) in the NMR structure is roughly similar to the corresponding segments of the self-associated Orai1 C-termini in the Hou *et al* Orai1 crystal structure, raising the possibility that the degree of conformational change in the Orai1 C-termini needed to accommodate STIM1 binding may be rather small instead of the complete extension suggested in the crystal structure study [24]. Thus, the nature of the rearrangements in Orai1 C-termini occurring during STIM1 binding differ in the two models.

To assess the nature and extent of conformational changes in the self-associated Orai1 C-termini during STIM1 binding, we introduced Cys residues at positions 273 and 276 to link the adjacent Orai1 coiled-coils via disulfide bonds. This strategy allowed us to examine the impact of cross-linking on STIM1-Orai1 association prior to and following STIM1 binding to the Orai1 C-termini. Our findings indicate that cross-linking the Orai1 C-terminus impairs STIM1’s ability to bind Orai1. Conversely, prior association of STIM1 with Orai1 diminishes the ability of the adjacent C-termini to cross-link. Further, using a combination of mutational analysis and cross-linking of a highly conserved SHK bend motif, upstream of the anti-parallel cross-over, we found that the bend is a crucial structural feature that plays an important role in setting up the Orai1 C-termini in the proper orientation to accommodate STIM1 binding. These results suggest that the coiled-coil motif seen in the crystal structure likely represents a quiescent state with only modest conformational changes needed for STIM binding.

## Experimental Procedures

### Cells and media

Human embryonic kidney cells from the HEK293 cell line were cultured at 37°C, 5% CO_2_ in suspension in CD293 medium (Life Technologies) supplemented with 4 mM GlutaMax (Life Technologies). In preparation for imaging and electrophysiology, at the time of passage cells were plated on poly-D-lysine-coated coverslips and grown in a medium containing 44% DMEM (Mediatech), 44% Ham's F12 (Mediatech), 10% fetal calf serum (HyClone), 2 mM glutamine, 50 U/ml penicillin and 50 μg/ml streptomycin until time of transfection, which occurred 24–48 hrs later after plating.

### Solutions and chemicals

The standard Ringer’s (2 mM Ca) solution used for FRET and confocal imaging studies contained the following (in mM): 155 NaCl, 4.5 KCl, 10 D-glucose, 5 Na-HEPES, 1 MgCl_2_ and 2 CaCl_2_. The Ca^2+^-free (0 Ca) Ringer’s solution contained 3 mM MgCl_2_, 1 mM EGTA (Sigma-Aldrich), and no added CaCl_2_. pH for both Ringer’s was adjusted to 7.4 with 1.0 N NaOH. Stock solution of ionomycin was respuspended in DMSO and used at the indicated concentration. Bis(2-mercaptoethylsulfone) (BMS) and diamide stock solutions were resuspended in 2 mM Ca Ringer’s solution and used at the indicated concentrations. For electrophysiology the standard 20 mM extracellular Ringer’s solution (20 Ca) contained the following (in mM): 130 NaCl, 4.5 KCl, 10 D-glucose, 1 MgCl_2_, 20 CaCl_2_, 10 mM TEA-Cl (prevents contamination from voltage-gated K^+^ channels), and 5 Na-HEPES, pH 7.4. The standard internal solution contained 135 mM caesium aspartate, 8 mM MgCl_2_, 8 mM BAPTA, and 10 mM Cs-HEPES, pH 7.2. All chemicals for Ringer’s solutions were obtained from Sigma-Aldrich with the exception of BAPTA which was purchased from Life Technologies.

### Plasmids and transfections

The Orai1–YFP plasmid has been previously described [23, 36]. STIM1-CFP, YFP–CAD, and CFP–CAD were kind gifts of Dr R. Lewis (Stanford University, USA). Unlabeled STIM1 used for the electrophysiology was obtained from Origene Technologies (Rockville, MD). Cysteine mutations were introduced at Orai1 residues L273 or L276 via site-directed mutagenesis using the QuickChange Mutagenesis Kit (Stratagene) and confirmed via DNA sequencing. FRET to assess binding between Orai1 and STIM1 (or CAD) was performed with YFP-labelled Orai1 constructs and STIM1-CFP or CFP-CAD.

For imaging studies, STIM1–CFP (or CFP–CAD), 100 ng, and the indicated Orai1-YFP, 100 ng, constructs were co-transfected using Lipofectamine 2000 (Life Technologies) and imaged 24 h later. Due to poor transfection efficiency of CFP-CAD, YFP-CAD was co-expressed with Orai1-YFP for Western experiments to ensure maximal CAD occupancy of the Orai1 channels. For electrophysiology experiments, unlabelled STIM1 (300 ng) was co-transfected with Orai-YFP (100 ng) constructs using Transpass D2 (New England Biolabs) and cells were patch-clamped 24 hrs later.

### Western Blot

HEK cells were treated with 0–500 μM diamide (Sigma-Aldrich) in 2 mM Ca Ringer’s for 20 minutes to induce the formation of disulfide bridges, then washed with cold PBS for 5 minutes. To quench unreacted thiols, cells were lysed in lysis buffer containing 50 mM N-ethylmaleimide (Sigma-Aldrich), 150 mM NaCl, 50 mM Tris, 1% Triton X-100, 0.1% SDS, and 1x protease inhibitor mixture (Roche) by incubating on ice for 15 min. Lysates were centrifuged at 4°C for 20 min and supernatants were collected. Isolated protein samples were heated to 65°C in Laemmli sample buffer (Bio-Rad) containing 0.1% mercaptoethanol and run on 4–20% SDS-polyacrylamide gradient gels (Bio-Rad). Protein was transferred onto nitrocellulose membrane and Orai1 was detected using 1:7500 purified monoclonal primary antibodies provided by Amgen (m266.1) [37] and 1:10000 peroxidase-labeled secondary antibody (GE Healthcare Life Sciences). GAPDH was blotted as a loading control. Monoclonal anti-GAPDH-Peroxidase (Sigma Alrdrich) antibody was the generous gift of the Volpert lab. Western blot images were analyzed using the image analysis software ImageJ. For CAD co-expression bots, WT-Orai1 and L276C-Orai1quantitations were quantitated at higher exposures due to their lower expressions compared to L273C-Orai1 channels under this condition. All conditions were quantitated at the highest exposure without saturation.

### FRET microscopy

HEK cells expressing Orai1-YFP and STIM1–CFP (or CFP–CAD) were imaged using wide-field epifluorescence microscopy on an IX71 inverted microscope (Olympus, Center Valley, PA, USA). Cells were imaged with a 60x oil immersion objective (UPlanApo NA 1.40), a 175W xenon arc lamp (Sutter, Novato, CA, USA), and excitation and emission filterwheels (Sutter). CFP, YFP and FRET images were captured at each timepoint using either a cooled CCD (Orca 285) or an EM-CCD camera (Hamamatsu, Bridgewater, NJ, USA) using optical filters specific for the three images as previously described [23]. Image acquisition and analysis was performed with Slidebook software (Imaging Innovations Inc., Denver, CO, USA). Images were captured at 12 s intervals at an exposure of 100 ms with an intensification of 225 and 1×1 binning (for the EM-CCD camera). Lamp output was attenuated to 25% by a 0.6 ND filter in the light path to minimize photobleaching. All experiments were performed at room temperature. Membrane expression of the Orai-YFP constructs was determined by examining the YFP intensity at regions of interest drawn around the PM from the YFP channel.

FRET analysis was performed using the FRET efficiency (E-FRET) method described by Zal & Gascoigne (2004) [38]. Bleedthrough factors (*a* = 0.1 and *d* = 0.39) were determined as previously described [23]. The apparent FRET efficiency was calculated from background-subtracted images using the formalism [38]:

$$\;Eapp=\frac{F_{C}}{F_{C}+GI_{DD}}$$

where, *F*_c_ = *I*_DA_-*aI*_AA_-*dI*_DD_

*I*_DD_, *I*_AA_ and *I*_DA_ refer to the background-subtracted CFP, YFP and FRET images, respectively. The instrument-dependent *G* factor was derived by measuring CFP fluorescence increase after YFP acceptor photobleaching using an intramolecular CFP-YFP fusion protein and had a value 1.19 ± 0.10. For each experiment, E-FRET values were plotted as a function of YFP/CFP ratios (acceptor/donor ratio) and analysis between different conditions was restricted to cells that had YFP/CFP values from 0.5–2.75, ensuring that E-FRET was compared across identical acceptor to donor ratios [38]. For steady-state measurements of CAD-Orai1 FRET or STIM1-Orai1 FRET, the E-FRET values were derived by drawing regions of interest (ROIs) around the PM. For timelapse CAD-Orai1 FRET or STIM1-Orai1 FRET, the analysis was obtained from ROIs drawn around the entire cell.

### Confocal microscopy

To show CAD co-localization with the various Orai1 mutants, HEK293 cells expressing CFP–CAD and the Orai1-YFP channels were imaged on a Fluoview FV10i confocal microscope (Olympus) equipped with a ×60 oil immersion objective. Cells were excited with 405 nm and 473 nm laser diodes and the intensity of laser light was attenuated to 50% for CFP (405 nm) and 15% for YFP (473 nm). Images were obtained at 1024×1024 pixel size at 4.5 s per frame and a slice thickness of 1.35 μm. The image acquisition parameters were kept constant across all experiments.

Confocal was also used to examine changes in CAD localization. For these experiments, HEK293 cells expressing YFP-CAD and Orai-CFP channels were used for easier visualization of CAD. Image acquisition parameters were identical to those described above. Images analysis was performed using NIH ImageJ software (NIH, Bethesda, MD). The cytosolic and plasma membrane pools of CAD were estimated from the mean intensities of YFP in regions of interest drawn around the PM and cytosol. The cytosol/membrane ratio of these values provided an estimate of the relative amount of CAD localized within the cytosol during the various treatments.

### Electrophysiology

Patch-clamp recordings were performed using an Axopatch 200B amplifier interfaced to an ITC-18 input/output board and an Apple computer as previously described [39]. Currents were filtered at 1 kHz with a 4-pole Bessel filter and sampled at 5 kHz. The holding potential was +30 mV unless otherwise indicated. The voltage stimulus consisted of a 100-ms step to -100 mV followed by a 100-ms ramp from -100 to +100 mV usually applied every 1 s. All data were corrected for liquid junction potential of the pipette solution and for leak currents collected in 50–150 mM LaCl_3_.

### Data Analysis

Bar graphs containing summary data are reported as the mean ± SEM. Two-tailed paired student t-test was used to compare between vehicle and diamide treatment conditions for a given construct unless otherwise indicated.

## Results

**Fig 2:**
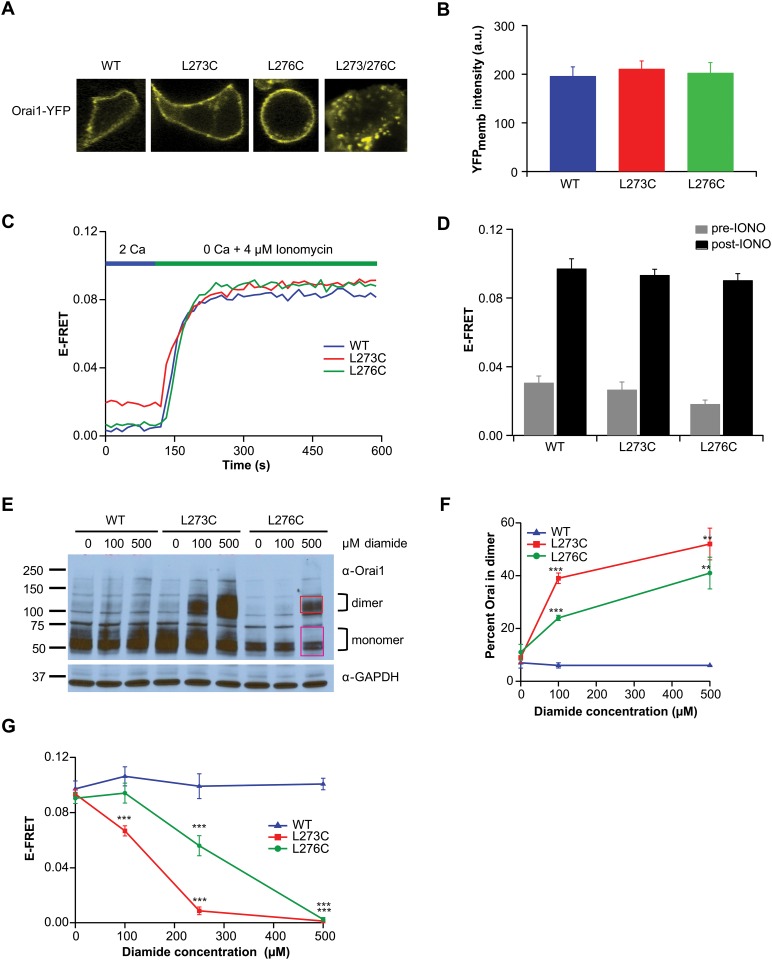
Cross-linking the Orai1 C-termini prevents STIM1 association. (A) Confocal images showing expression of the Orai-YFP single and double cysteine mutants at residues L273 and L276. Whereas the single L273C and L276C mutants are expressed in the plasma membrane, the L273/276C double mutant was only found in unknown intracellular compartments. Cells were transfected with the indicated Orai1 constructs together with CFP-CAD. (B) Summary of Orai-YFP fluorescence at the plasma membrane analyzed from widefield YFP images acquired from cells transfected with CFP-CAD and the indicated Orai1-YFP constructs. Data points are mean ± SEM of 23–26 cells. (C) Representative traces showing E-FRET changes following store depletion by 4 μM ionomycin in single HEK293 cells co-expressing STIM1-CFP and the indicated Orai1-YFP proteins. (D) Summary of E-FRET values of the Orai1 channels before and after store depletion by ionomycin (IONO). For each individual cell, the E-FRET value was averaged from three frames. Data points are mean ± SEM of 21–48 cells. The post-ionomycin E-FRET values of the L273C and L276C channels are not significantly different than that of WT Orai1 channels. (E) Western blot of cell lysates expressing WT Orai1-YFP, L273C Orai1-YFP, or L276C Orai1-YFP channels exposed to varying concentrations of diamide (0–500 μM). Boxes drawn in the L276C Orai1-YFP 500 μM treatment lane indicate the regions used for the quantitation of monomer (magenta) and dimer (red) values. (F) Western blot quantitation of dimer formation at various diamide concentrations (0–500 μM). The percent of total Orai1 cross-linked was calculated as dimer/(monomer + dimer). Data points are mean ± SEM for 4–7 cells (*** p ≤ 0.001; ** p ≤ 0.01). (G) Steady-state E-FRET values of cells co-expressing Orai1-YFP channels with STIM1-CFP and pre-treated with various concentrations of diamide (0–500 μM) prior to store-depletion. Data points are mean ± SEM for 12–51 cells (*** p ≤ 0.001).

The recent elucidation of the *Drosophila* Orai1 crystal structure [24] revealed an unexpected structural feature of the cytosolic C-terminal tails. The C-termini of adjacent monomers were observed to be self-associated through an anti-parallel coiled-coil arrangement mediated by hydrophobic interactions between the *Drosophila* equivalents of L273 (I316) with L276 (L319). The presence of this Orai1 C-terminal coiled-coil unit raises the possibility that the relevant STIM1 binding interface is the self-associated unit rather than individual Orai1 C-termini. To examine whether the coiled-coil unit could act as a binding interface, exogenous cysteines were introduced at these two residues to stabilize their association through disulfide cross-linking. We found that the L273C and L276C single mutants expressed well in the plasma membrane (Fig 2A) and interacted with STIM1 at levels similar to WT Orai1 as assessed by FRET (Fig 2B–2D). However, the L273/L276C double mutant appeared to be expressed primarily in unknown intracellular organelles and therefore was not used in this study (Fig 2A).

### Cross-linking of Orai1 C-termini impairs STIM1 association

Given the close proximity of the L273 residues in the two protomers we reasoned that the single cysteine mutants could be used to promote a homotypic (L273C-L273C) disulfide bond to stabilize the self-association of the adjacent anti-parallel C-termini. Likewise, cross-linking of the nearby L276C residues in the adjacent monomers could also fix the self-association of the Orai1 C-termini. To test this idea, HEK293 cells expressing either WT-YFP or L273C-YFP or L276C-YFP single mutants were treated with varying concentrations of the oxidizing reagent, diamide, and disulfide bond formation was assessed by Western blot (Fig 2E). WT Orai-YFP was observed to exist largely as a monomeric species (~61 kDa) at all concentrations of diamide treatment. By contrast, L273C-YFP and L276C-YFP showed a dose-dependent increase in Orai dimers (~122 kDa) (Fig 2E and 2F). Because we used relatively low concentrations of the oxidizing agent (0–500 μM), we assume that disulfide bond formation occurs within the self-associated Orai1 C-termini. Interestingly, L276C cross-linked less efficiently than L273C at all concentrations of diamide tested. We suspect that this lower ability of L276C to form diamide induced disulfide bonds is likely due to the greater distance between the side chains of the L319 pairs, (*Drosophila* equivalent of human Orai1 L276; C_β_s are 6.9 angstroms apart) compared to the distance of the I316 pairs (human Orai1 L273 equivalent; C_β_s are 6.0 angstroms apart) as revealed in the dOrai crystal structure [24]. These data indicate that the residues L273 and L276 in the adjacent protomers are located close enough to form homotypic disulfide bonds in the cysteine mutants.

Having established that these single mutants were linkable via disulfide bonds, we next assessed the functional consequences of this self-association on STIM1-Orai1 binding by FRET (Fig 2G). For this test, cells co-expressing Orai1-YFP (WT, L273C, or L276C) and STIM1-CFP were first exposed to diamide to promote formation of homotypic disulfide bonds. The ionophore ionomycin was then used to deplete ER calcium stores, thereby forcing STIM1-CFP movement to ER-PM junctions where it would be available to bind to Orai1-YFP. We found that for both residues, STIM1-Orai1 association was almost completely disrupted by 500 μM diamide, which produced roughly 52% and 41% cross-linking of L273C Orai1-YFP and L276C-YFP, respectively, as assessed by Western blot (Fig 2G). Since WT Orai1-YFP is unaffected by diamide treatment, the inhibition of STIM1-Orai1 FRET seen in the L273C and L276C Orai1 mutants can be attributed to the exogenous cysteines introduced at these residues. Consistent with the lower ability of L276C residues to cross-link with each other, L276C-Orai1 did not show reduced STIM1 binding until 250 μM diamide was administered, while at this same concentration of diamide, STIM1-CFP/Orai1-YFP FRET was nearly completely inhibited in L273C Orai1. The inhibition of STIM1-Orai1 association by cross-linking the Orai1 C-termini prior to STIM1-binding indicates that fixing the self-association of the Orai C-termini is detrimental for STIM1 binding and suggests that at least some rearrangement of the C-termini is required for STIM1 interaction.

### Cross-linking efficiency of L273 and L276 is regulated by STIM1 binding

**Fig 3:**
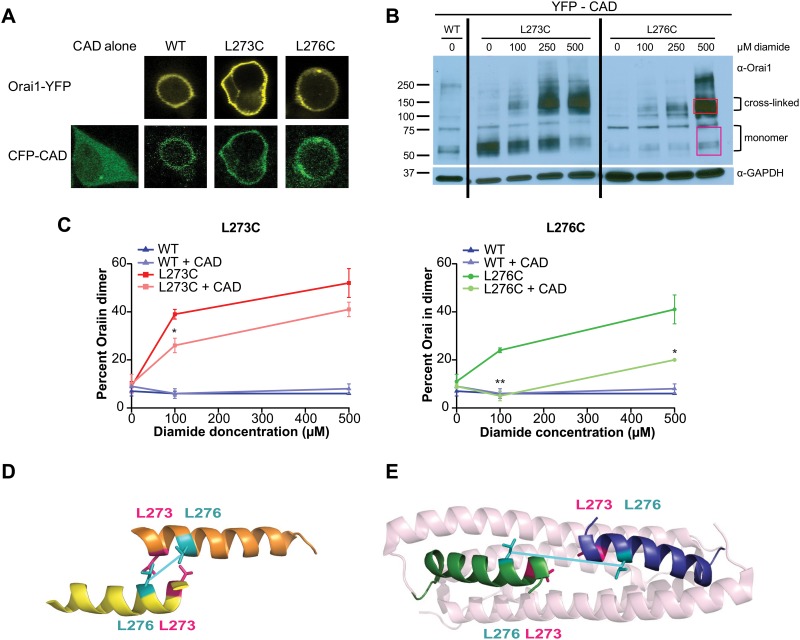
State dependence of cross-linking: prior CAD binding impairs Orai C-terminal self-association. (A) Confocal images showing CFP-CAD co-localization with WT and mutant Orai1-YFP channels. (B) Western blot of cell lysates co-expressing YFP-CAD with WT Orai1-YFP, L273C Orai1-YFP, or L276C Orai1-YFP channels exposed to varying concentrations of diamide (0–500 μM). The WT-Orai1 and L276C-Orai1 lanes show blot that was exposed for 5 minutes, while the L273C-Orai1 blot, which showed higher protein expression, was exposed for only 30 seconds. The complete blot for each exposure is shown in S1 Fig. (C) Western blot quantitation of dimer formation at various diamide concentrations (0–500 μM) for L273C (left) and L276C (right) in the presence and absence of YFP-CAD. The percent of total Orai1 cross-linked was calculated as dimer/(monomer + dimer). Data points are mean ± SEM for 3–7 cells. Statistics in these graphs represent significance between diamide treatments in the presence and absence of YFP-CAD (** p ≤ 0.01; * p ≤ 0.05) (D and E) Comparison of positioning of the L273 (pink) and L276 (cyan) side chains in the NMR complex structure (Orai helices are in blue and green, the STIM1 fragments are represented in light pink) (PDB ID 2MAK) and the positions of the *Drosophila* equivalents I316 (hot pink) and L319 (cyan) in the *Drosophila* crystal structure (helices shown in yellow and orange) (PDB ID 4HKR). PyMol mutagenesis wizard was used to substitute a leucine residue at position 316 which is an isoleucine in the *Drosophila* structure.

In the experiments described above, cross-linking of Orai1 subunits was elicited in resting cells with replete ER Ca^2+^ stores. To understand whether the adjacent L273 and L276C residues can be cross-linked even when STIM1 is already bound to Orai1, we next examined disulfide bond formation in HEK cells co-expressing Orai1 with soluble CAD (Fig 3A–3C), which directly binds and activates Orai1 without requiring Ca^2+^ store-depletion [17–19]. In both mutants, diamide induced the formation of Orai1 dimers in the presence of YFP-CAD (Fig 3B and 3C). However, as seen when Orai1 was expressed alone (Fig 2E and 2F), L273C formed disulfide bonds more readily than L276C even in the presence of CAD. For example, in the presence of CAD, 500 μM diamide evoked ~41% dimerization in L273C Orai1 compared to only ~20% for L276C Orai1 (Fig 3C).

Importantly, comparison of dimer levels in the presence and absence of CAD revealed that the extent of Orai1 disulfide bond formation is markedly lower when CAD was co-expressed (Fig 3C). Thus, prior CAD binding appears to impede the ability of cysteine pairs at both positions to form disulfide bonds. In the NMR complex structure, both L273 and L276 are engaged in interactions with the STIM1 fragment and could therefore explain the decreased cross-linking efficiency at the residues [35]. However, the state-dependence of disulfide bond formation was observed to be more drastic for the L276C-YFP mutant (Fig 3B and 3C). For example, at 500 μM diamide CAD co-expression reduced the number of observed dimers by ~21% for L273C while the there was a ~51% reduction in observed dimers for L276C with CAD (Fig 3C). Collectively, the reduction in homotypic cross-linking caused by CAD binding indicates that L273 and L276 pairs move further away from themselves following STIM1 binding. The greater decline in L276 cross-linking further implies that the L276 residue pairs undergo a larger shift than the L273 residue pairs (Fig 3D and 3E).

**Fig 4:**
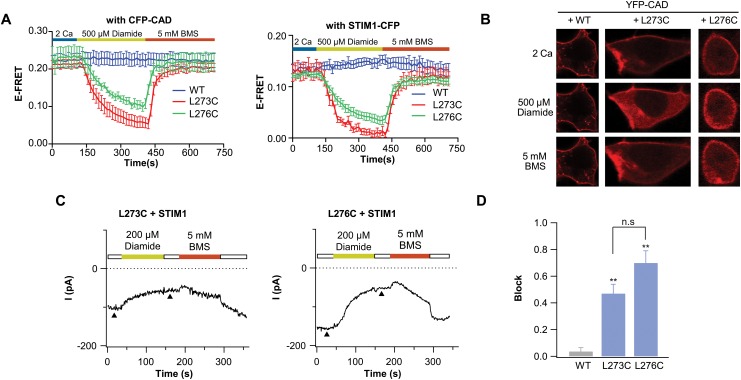
Induction of disulfide bonds in STIM1 bound channels diminishes STIM1 association and I_CRAC_. (A) E-FRET traces of cells co-expressing the indicated Orai1-YFP constructs with CFP-CAD (left) or STIM1-CFP (right). Cells were treated with 500 μM diamide followed by 5 mM BMS. Data points are mean ± SEM for 4–8 cells. (B) Confocal images showing CFP-CAD localization of cells co-expressing CFP-CAD with the indicated Orai1-YFP channels treated with 500 μM diamide then 5 mM BMS. (C) Time-course of Orai1 currents from cells co-expressing STIM1 with the indicated Orai1-YFP channels. After development of a stable current, 200 μM diamide was applied to induce Orai1 cross-linking. This was followed by application of 5 mM BMS as indicated. The diamide induced inhibition of Orai1 current is largely restored following administration of BMS. Note that because BMS itself slightly blocks I_CRAC_ [40], the full extent to relief by BMS requires its washout. Arrowheads denote the time points where current amplitudes were analyzed for block (D) Summary of block produced by 500 μM diamide. Data points are mean ± SEM for 4 cells (** p ≤ 0.01).

In principle it is possible that the decrease in cross-linking seen in the Westerns where CAD and Orai are co-expressed is the result of cross-linking of a small fraction of channels that are not bound to CAD. To determine if this is the case, we examined the effects of acute applications of diamide over the course of a timelapse E-FRET experiment to monitor changes in CAD-Orai1 binding in real-time (Fig 4A). Diamide treatment led to a decline in CAD-Orai1 association in both the L273C and L276C mutants, which was not observed in wild-type Orai1 (Fig 4A, left, blue trace), indicating that the decline is specific to the exogenous cysteines. Consistent with the loss of CAD-Orai binding, examination of CAD localization via confocal microscopy revealed that cytosolic YFP-CAD localization increases upon treatment with diamide in L273C-Orai1. Conversely, administration of BMS to reduce disulfide bonds at the Orai1 C-terminus, rapidly restored CAD-Orai association as monitored by FRET or by the localization of CAD at the plasma membrane (Fig 4A and 4B). Together, these results indicate that diamide induced cross-linking of Orai1 C-termini disrupts CAD binding.

In agreement with the results of the Western blots indicating that L276C cross-links to a smaller extent than L273C (Figs 2 and 3), FRET experiments showed that L276C Orai1-STIM1 association is inhibited to a smaller extent compared to L273C Orai1-CAD association following diamide application (Fig 4A, right). A similar trend was observed when the experiment was conducted with full-length STIM1, although, here the extent of E-FRET decline was greater for both mutants (Fig 4A, right). We suspect that the greater decline in E-FRET seen with STIM1 (compared to CAD) may be due to the fact that the expression of full-length STIM1 is likely lower than that of soluble CAD. As a result, the binding sites on Orai1 may likely be saturated with CAD over-expression, but not with STIM1 over-expression. Collectively, these results indicate that disulfide bond driven self-association of the Orai1 C-termini and STIM1-binding are mutually exclusive.

We next examined the effect of cross-linking the C-termini on channel activity. In order to observe changes in channel function due to the disulfide linking of L273C or L276C, pairs diamide was applied to channels already activated by STIM1 in store depleted cells. STIM1 activated Orai1 currents produced by L273C and L276C both showed inhibition following administration of 200 μM diamide (Fig 4C and 4D), which could be reversed by 5 mM BMS. Puzzlingly, in contrast to effects on STIM1 binding, the extent of current decline was greater in L276C Orai1 compared to L273C Orai1. We do not know the mechanistic basis of this difference, but it could be related to additional effects of L276 for channel gating. These results indicate that cross-linking of the Orai1 C-termini results in abrogation of CRAC channel activity, most likely due to impaired Orai1-STIM1 binding. Taken together with the E-FRET results, these data indicate that STIM1 binding is accompanied by rearrangements in the anti-parallel coiled-coil C-terminal.

#### An “SHK” bend is crucial for setting up the correct conformation of the C-terminus for STIM1 binding

**Fig 5:**
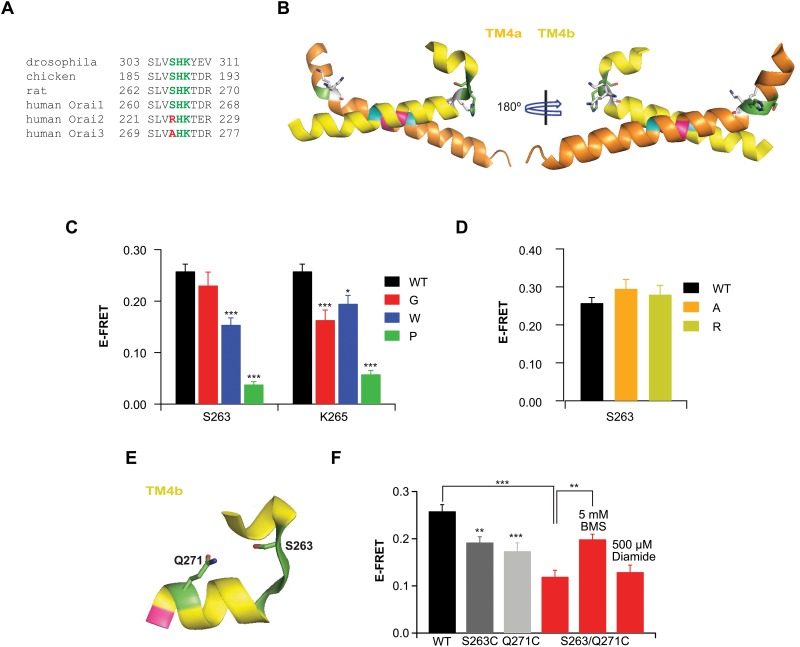
A conserved motif in the bend region of the Orai1 C-terminus is required for STIM1 binding. (A) Sequence alignment of the Orai1 C-terminus from various species shows a conserved stretch of amino acids spanning residues 260–268 (human Orai1) that include the SHK residues. (B) Close-up of *Drosophila* Orai1 C-terminus with the conserved ‘SHK’ bend highlighted in green. Because the side chain for K265 was not modeled into the *Drosophila* structure, we used the PyMol mutagenesis wizard to generate three different potential rotomer conformations of this residue. (C) Summary of E-FRET for mutants of S263 and K265 Orai1-YFP with CFP-CAD. E-FRET values for each cell were averaged from three frames. Data points are mean ± SEM for 15–42 cells (*** p ≤ 0.001; * p ≤ 0.05). (D) Summary of E-FRET for mutation of S263 in Orai1-YFP to the equivalent residues in Orai2 (arginine, R) and Orai3 (alanine, A) with CFP-CAD. Data points are mean ± SEM for 16–19 cells. (E) Close-up of TM4a ‘SHK’ hairpin bend in one monomer. Highlighted in green are residues S263 and Q271, which were mutated to cysteine. Formation of an intra-monomer disulfide bond between these residues is predicted to restrict conformational changes at the bend. (F) Summary of E-FRET between the S263/Q271C Orai-YFP bend double mutant and CFP-CAD, in the presence of diamide or BMS. E-FRET values for each cell were averaged from three frames. Data points are mean ± SEM for 15–32 cells (*** p ≤ 0.001; ** p ≤ 0.01).

Within the Orai1 cytosolic C-terminus, lies a conserved stretch of amino acids _260_SLVSHKTDR_268_ of which three residues (S263/H264/K265) are located at the bend in the C-terminus and appear to aid the formation of the unique anti-parallel coiled-coil of the adjacent termini (Fig 5A and 5B). This “SHK” motif is unusual in that it only slightly bends one C-terminus but permits the adjacent C-terminus to completely bend back and change directions (Fig 5B). The recent NMR solution structure of a complex containing a human STIM1 fragment (amino acids 312–387) with a human Orai1 C-terminal fragment (amino acids 272–292) shows that the C-terminal fragments are in an orientation comparable to that observed in the *Drosophila* crystal structure [35]. Therefore, we reasoned that this motif may likely play an important structural role in maintaining the C-termini in the proper orientation for human Orai1 as well. To test this idea, a series of mutations predicted to disrupt the “SHK” bends were made at S263 and K265 and tested for their ability to bind CFP-CAD (Fig 5C). At S263, a mutation to glycine showed no signification alteration of CAD-Orai1 association as assessed by FRET. A more dramatic mutation to tryptophan, which may cause steric hindrance with side chains of neighboring residues, diminished the Orai1-CAD binding. Likewise, a substitution to proline, which is expected to distort or kink the helix, also significantly diminished the mutant Orai1’s ability to interact with CAD. Patch-clamp recordings showed that the S263P mutation also dramatically impaired store-operated Orai1 currents (S2 Fig). Mutations at K265, however, failed to show a pattern. Here, even a glycine substitution significantly reduced CAD-Orai1 association to levels comparable to the tryptophan mutation. Introduction of an exogenous proline, which would be expected to distort or kink the helix, impaired CAD binding even more dramatically. Patch-clamp analysis showed that this Pro mutation also severely diminished Orai1 currents (S2 Fig). Although we do not know the extent of the structural disruption that these mutations elicit, it is highly likely that the bulky tryptophan and the proline substitutions would alter the relative orientations of the two C-termini from that depicted in the crystal structure. Together, these data indicate that mutations at the SHK bend predicted to disrupt the motif negatively impact CAD’s ability to associate with Orai1.

In Orai2 and Orai3, the serine at 263 is replaced by either arginine or alanine (resulting in the amino acid sequences RHK and AHK, respectively). To explore the functional effects, if any, of this change, we mutated S263 to either arginine or alanine and examined the functional consequences for CAD binding. FRET analysis showed that S263R and S263A Orai1-YFP mutants interacted with CFP-CAD at levels comparable to WT Orai1 (Fig 5D), suggesting that the overall structure of the bend is probably similar in the three Orai isoforms.

The probable structural flexibility of the “SHK” motif led us to next consider whether this domain contributes to the conformational rearrangements that allow the Orai C-termini to accommodate STIM1 binding. If STIM1 binding requires alterations in the configuration of the SHK bend motif, we reasoned that constraining flexibility at this location could impair STIM1-Orai1 association by preventing the lateral displacement of the L273 and L276 pairs. To test this idea, two exogenous cysteines were introduced on opposite ends of the bend, at residues S263 and Q271, with the expectation that induction of disulfide bonding between the two residues would prevent conformational rearrangements (Fig 5E). The S263C-YFP and Q271C-YFP single mutants showed robust, albeit modestly reduced association with CFP-CAD (Fig 5F). However, the S263/Q271C-YFP mutant showed a more significant drop in E-FRET compared to WT Orai1. Application of diamide had no impact on association with CAD. However, the diminished CAD-Orai1 association could be rescued to single mutant levels of interaction with CAD by application of BMS. These results suggest that the two cysteines likely formed disulfide bonds even in the absence of diamide, and BMS removes this link to rescue CAD binding (Fig 5F). Importantly, the recovery of CAD binding by administering BMS suggests that flexibility at the “SHK” motif is required to allow optimal STIM1 binding at the Orai1 C-terminal site.

## Discussion

The interaction of STIM1 with the C-terminus of Orai1 (amino acids 268–291) is believed to occur via the formation of coiled-coils between Orai1 and the CAD domain [24, 35]. The structural and molecular basis of this interaction, however, remains incompletely understood. The recent elucidation of a hexameric *Drosophila* Orai channel has revealed that the hexamer can in essence be broken down into a trimer of dimeric Orai subunits. Although, six-fold symmetry is maintained throughout the transmembrane helices, the cytosolic extensions of TM4 depart from this symmetry. This departure of symmetry is the result of these extensions associating with each other in an anti-parallel coiled-coil [24]. The contribution of this motif to STIM1-Orai1 association is currently under debate. One proposed model hypothesizes that the anti-parallel coiled-coil observed in the crystal structure dissociate and the individual Orai C-termini extend into the cytosol to interact with STIM1 [24]. However, the recent NMR complex structure between STIM1 (amino acids 312–387) and Orai1 (amino acids 272–292) fragments suggests an alternative model wherein the Orai1 C-termini remain in a similar orientation to the coiled-coil form of the *Drosophila* crystal structure, but with some minor alterations in the angles of the Orai1 helices [35]. Our findings here suggest STIM1 binding leads to a conformational change of the C-termini but they are likely to retain a similar anti-parallel orientation.

The *dOrai* structure reveals that the anti-parallel associated configuration of the Orai C-termini is set up by a flexible bend within the C-terminus between amino acids 263–265 [24]. We used a combination of mutagenesis and cross-linking to examine the structural importance of the “SHK” bend motif upstream of the L273-L276 contact site, in setting up the C-termini configuration seen in the crystal structure. Mutations that are expected to alter the orientation of the bend negatively impacted STIM1-Orai1 association and CRAC channel function (Fig 5C). Interestingly, clamping the bend via intrasubunit cross-linking impaired, but did not completely prevent STIM1 binding (Fig 5F). These results suggest that flexibility is required at this “SHK” motif to accommodate STIM1 and that configuration of the bend plays an important role in STIM1-Orai1 association.

To examine the functional importance of the self-association of the Orai C-termini for STIM1 binding we introduced cysteine residues at the contact sites within the C-terminal anti-parallel coiled-coil, at residues L273 and L276, and linked the two subunits by disulfide bond crosslinks (Fig 1C). Interestingly, our data point to a differential ability to induce cross-links at the chosen sites with L273C pairs showing more efficient cross-linking than L276C pairs (Fig 2E and 2F). This residue specific difference is compatible with the increased distance between the L319 residue pairs (the *Drosophila* equivalent of L276 residues) observed in the crystal structure [24] thus supporting the existence of the anti-parallel motif *in situ*. Functionally, we find that cross-linking the C-termini together in resting cells prevents STIM1 from binding to Orai1 following subsequent depletion of ER Ca^2+^ stores (Fig 2G). This suggests that the forced self-association likely prevents a necessary structural change in the Orai1 C-termini that occurs during STIM1 binding. More intriguingly, cross-linking the Orai1 C-termini when STIM1 or CAD are pre-bound to Orai1 also disrupted STIM1-Orai1 binding, consistent with the idea cross-linking and STIM1 binding are mutually exclusive (Fig 4A and 4B).

The diminished ability to cross-link L273C and L276C pairs when CAD is pre-bound to the channel is consistent with the idea that cross-linking stabilizes the STIM1-free state of the channel and that STIM1 association induces a conformational change that impedes self-association of the Orai1 C-termini (Fig 3B and 3C). Our data cannot distinguish whether cross-linking of the Orai1 C-termini occurs in the CAD-bound state of the channel with cross-linking resulting in the unbinding of CAD from Orai1, or if CAD first had to pop off before the cysteines can cross-link, with cross-linking preventing CAD rebinding to Orai1. However, recent evidence suggests that the interaction between STIM1 and Orai1 is rather labile [16, 35] so we favor the idea that the formation of disulfide bonds occurs following CAD unbinding and the now linked C-termini no longer bind CAD effectively. Moreover, the available NMR complex structure between human Orai1 and STIM1 fragments suggests that both L273 and L276 are located at or very close to the binding interface with STIM1 [35]. Hence, lack of cross-linking in the presence of CAD could arise simply because of the residues are unavailable for modification.

What are the implications of the differential cross-linking of L273C and L276C in the presence of CAD for the nature of the rearrangements at the C-terminus following STIM1 binding? We explore this in context of the available NMR complex structure containing two Orai1 C-terminal fragments (amino acids 272–292) that maintain an anti-parallel orientation while bound within identical pockets on either end of the STIM1 dimer (amino acids 312–387) [35]. Both Orai fragments are involved in heterotypic coiled-coil interactions with the CC2 domain of CAD (amino acids 363–389). However, comparison to the orientation of these Orai regions in the crystal structure suggests that in the STIM1-bound state, the helices have shifted relative to each other such that the L273 and L276 residues of the adjacent monomers no longer form contacts with each other (Fig 3D and 3E) [35]. The supercoiling of the Orai1 fragment has also changed in a way that positions L273 and L276 now sit on opposite faces of the helix (Fig 3E). As a consequence of these changes, the L276 pairs have a greater lateral separation (C_β_s are 20.1 angstroms apart) in the STIM1-bound state compared to the L273 pairs (C_β_s are 9.7 angstroms apart). Although, our methodology cannot determine the exact positioning of the residues when STIM1 is bound, the diminished cross-linking in the presence of CAD is compatible with the distance differences observed for the residues in the NMR complex [24, 35]. Together, these results support a model wherein the self-associated Orai1 C-termini rearrange only modestly to accommodate STIM1 binding, in a way that preserves the overall orientation of the Orai1 C-termini as observed in the STIM1/Orai1 fragment NMR structure.

## Supporting Information

S1 Fig Cross-linking of CAD-bound channels. Due to lower expression of WT-Orai1 and L276C-Orai1 compared to L273C-Orai1, higher exposures were needed to visualize and quantitate cross-linking. The blot depicted in Fig 3 is shown here at both 30 s (top) and 5 min. (bottom) exposures.
(EPS)

S2 Fig Patch-clamp analysis of “SHK” proline mutations. (A) Plot of peak current amplitudes measured during steps to -100 mV for WT, S263P, and K265P co-expressed with STIM1. Each point shows the mean ± of 2–4 cells. (B) Plots of the I-V (current-voltage) relationship for WT, S263P, and K265P Orai1 channels with STIM1 in 20 mM Ca Ringer’s solution.
(EPS)
